# Acute Parastomal Hernia Presentations: A 10-Year Review of Management and Outcomes

**DOI:** 10.3389/jaws.2024.13364

**Published:** 2024-11-28

**Authors:** Raziqah Ramli, Zi Qin Ng, Jason Diab, Andrew Gilmore

**Affiliations:** ^1^ Department of Colorectal Surgery, Liverpool Hospital, Liverpool, NSW, Australia; ^2^ School of Medicine, University of New South Wales, Sydney, NSW, Australia; ^3^ Department of Surgery, Macquarie University Hospital, Sydney, NSW, Australia

**Keywords:** parastomal hernia repair, parastomal hernia, incisional hernia repair, emergency hernia surgery, emergency parastomal hernia

## Abstract

**Introduction:**

The acute presentation of parastomal hernia (PSH) can range from exacerbation of pain to life-threatening incarceration. Managing the acute PSH is challenging, particularly in the presence of concomitant midline incisional hernia. Most literature focuses on the outcomes of elective PSH repair. There is a paucity of literature on optimal management approaches to emergency PSH presentations. We aim to evaluate the outcomes of management of acute PSH presentations at a large acute tertiary hospital over a 10-year-period.

**Methods:**

A retrospective analysis performed from May 2013 – May 2023 for all acute parastomal hernia presentations. The data collated included: demographics, index operation/pathology, duration of the stoma, clinical presentation, laboratory and imaging results and management outcomes (non-operative vs. operative intervention).

**Results:**

Twenty-two admissions of acute PSH over the study period with the median age of 77 years, and 14 males. The median Charlson comorbidity score was 5. Most patients had stoma formation due to malignancy (12) with most end-colostomy (10). 11 patients had previous PSH repairs. 13 patients underwent operative intervention on index presentation via a combination of approaches. 4 required small bowel resection and 4 had resection of stoma; 4 had relocation of the stoma. There was one postoperative death due to sepsis related multi-organ failure. There were five recurrences of PSH on follow-up. Of the nine patients managed non-operatively, seven subsequently had elective reconstruction.

**Conclusion:**

Acute PSH presentation usually requires operative intervention with considerable recurrence rates. The approach to the PSH repair, in the acute setting, needs to be individualised. Further study is required to assist with the development of guidelines for managing this difficult problem.

## Introduction

Parastomal hernia (PSH) occurs commonly after the formation of an ostomy [1]; with an incidence reported up to 80% [2]. The incidence of recurrent PSH, after repair, is up to 63% [3]. Most published literature focus on different techniques, and outcomes, following elective PSH repair. The last decade witnessed an increased interest in prophylactic mesh placement during the index surgery to prevent occurrence of PSH [4, 5]. Despite this interest, data from recent studies on prophylactic mesh placement PSH is disappointing [6]. Notably, literature is sparse when it comes to the management of acute presentation of PSH. Often, this group of patients also suffer from concomitant midline incisional hernias which add to the complexity of decision making. A recent study from the USA, utilising the Medicare claims, reported high morbidity associated with emergency PSH repair [7]; but did not comment on patients with concurrent midline incisional hernias. Presently, there are no guidelines to aid decision making for optimal management of acute PSH presentation.

This study aims analyses the management and outcome of emergency presentation of parastomal hernia over a 10-year period, at a large Australian Acute Care Tertiary Hospital.

## Methods

A retrospective review performed over a 10-year period from March 2013 to March 2023 on all consecutive acute presentations of PSHs. Ethics approval was obtained from the Human Research Ethics Committee (HREC) with approval number ETH02345. Only patients who presented to the Emergency Department with acute parastomal hernia diagnosed clinically or with imaging were included. Patients who were undergoing elective parastomal hernia repairs were excluded. Detailed data on each emergency PSH admission was collected regardless of re-admission of the same patient. The data collected included: patient demographics, Body Mass Index (BMI), type of stoma, reason for initial stoma formation, age of stoma, number of previous parastomal repair, symptoms (pain, vomiting, reduced stoma output), days of symptoms before presentation, Charlson Comorbidity Index (CCI) score [8], and common risk factors in patients. Blood results obtained included white cell count (WCC), C-reactive protein (CRP), creatinine, estimated glomerular function (eGFR) and lactate. Computed tomography (CT) scan results collated included PSH contents and presence of midline herniae using the European Hernia Society (EHS) Classification [9]. If patient underwent non-operative management, data collection included analgesia, dexamethasone and/or the use of nasogastric decompression with other non-operative adjucts. If patients underwent operative management; data collected included operative approach (open - midline or parastomal (circumferential stomal incision), laparoscopic, and hybrid), need for resection of small intestine, or, ileal/colonic conduit, re-siting of ostomy (including location), use of mesh and type, the use of Botulinum and the need for component separation. Length of stay (LOS), LOS in intensive care unit, death, recurrence of PSH and other complications recorded. Patients undergoing subsequent elective repair, after admission, also determined. Complications were reported as per the Claven-Dindo classification system [10].

### Statistical Analysis

Statistical analyses were performed with the SPSS Software Package (IBM SPSS Statistics for Windows, Version 21.0. Armonk, NY). Normally distributed data were presented as means with standard deviation (SD), while non-parametric data were presented as medians with interquartile ranges (25th percentile - 75th percentile value) or as means with standard deviations (SDs). Categorical variables were presented as numbers with percentages (%).

## Results

There were 22 admissions from 19 patients during the study period; 12 males and 7 females. The median age 77 years (range 65–82 years). The median BMI was 29 (range 27–34). The median Charlson comorbidity score was 5. 11 patients had hypertension, 9 patients had hypercholesterolemia, 8 had diabetes mellitus, 3 had obstructive sleep apnea and 2 patients had COPD. The median duration since stoma formation was 9 years (range 3–7 years). 6 stomas were formed laparoscopically and 13 were formed via open surgery. 12 patients had stoma formed for malignancy, four for inflammatory bowel disease, two from sepsis and two from incontinence (Table 1).

**TABLE 1:** Demographics of patients.

Variable	N (%)	Details
Age (years)	77 (69–82)^a^	
Sex (male)	14 (64)	
Body Mass Index (BMI)	29 (27–34)^a^	
Charlson comorbidity index	5 (3–7)^a^	
Risk factors		
Diabetes	8 (36)	
Smoking	8 (36)	
Hypertension	11 (50)	
Hypercholesterolemia	9 (41)	
Chronic Obstructive Pulmonary Disease (COPD)	2 (9)	
Obstructive Sleep Apnea	3 (14)	
Duration since stoma formation (years)	9^a^ (5–15)	
Laparoscopic stoma formation	6 (32)	
Open stoma formation	13 (68)	
Reason for initial stoma		
Malignancy	12 (55)	
Inflammatory Bowel Disease	4 (18)	
Sepsis	4 (18)	Necrotising fascilitis (n = 1), Colonic perforation (n = 1), Colovesical fistula (n = 1), strangulated richter’s hernia (n = 1)
Incontinence	2 (9)	
Type of stoma		
End Colostomy	10 (46)	
Ileal urinary conduit	4 (18)	
End colostomy and ileal conduit	4 (18)	
Loop colostomy	2 (9)	
Loop ileostomy	1 (5)	
Previous repair of parastomal hernia	11 (50)	
Number of previous repair:		
1	10 (46)	
4	1 (5)	

^a^ median, (IQR, 25th percentile-75th percentile).

Of the ostomies, 10 were end colostomy, four were ileal conduit, four both end colostomy and ileal conduit, two loop colostomy and one loop ileostomy.

Eleven patients had prior PSH repair before admission, with one patient having four previous PSH repairs and the remaining patients having only one previous repair.

Patients had a median of 1 day of symptoms before presentation. All patients presented with pain, while 12 had vomiting and 18 had reduced stoma output. Biochemical markers of patients on presentation showed a median eGFR of 67 mL/min/1.73 m^2^, creatinine of 86(μmol/L), lactate of 1 mmol/L, white cell count of 9 × 10^9/L and C-reactive protein level of 11 mg/L (Table 2).

**TABLE 2:** Patient clinical presentation and biochemical markers.

Variables	N (%)
Length of hospital stay (days)	7 (3–17)^a^
Day(s) of symptoms	1 (1–2)^a^
Presenting symptoms	
Pain	22 (100)
Vomiting	12 (55)
Reduced stoma output	18 (81)
Biochemical markers	
Estimated Glomerular Filtration Rate (mL/min/1.73m^2^)	67 (46–85)^a^
Creatinine (μmol/L)	86 (72–124)^a^
Lactate (mmol/L)	1 (0–2)^a^
White Cell Count (x 10^9/L)	9 (7–16)^a^
C-reactive protein (mg/L)	11 (2–34)^a^

^a^ median, (IQR, 25th percentile-75th percentile).

13 patients underwent operative management during the index admission and nine were managed non-operatively. A total of six surgeons were involved in the care of these patients. The PSH contents were classified via the EHS classification [9], 14 patients with type I, two with type II, three with type III and three with type IV (Table 3). There were five patients with concomitant midline hernia.

**TABLE 3:** Description of contents of hernia and management.

Variables	N (%)
Findings	
I (<5 cm defect with no concomitant incisional hernia)	14 (64)
II (<5 cm defect with concomitant incisional hernia)	2 (9)
III (>5 cm defect with no concomitant incisional hernia)	3 (14)
IV (>5 cm defect with concomitant incisional hernia)	3 (14)
Management	
Operative management on index admission	13 (59)
Non-operative management	9 (41)

Out of the nine patients managed non-operatively, six had nasogastric tube decompression and only two patients out of these received intravenous dexamethasone dose of 8 mg, with one patient had a fleet enema delivered via placement of a urinary catheter into the stoma. The remaining three patients were not obstructed and were only managed with analgesia for pain (Table 4).

**TABLE 4:** Description of Non-operative techniques.

Variables	N (%)
Analgesia only for pain relief	3 (14)
Nasogastric decompression only	3 (14)
Nasogastric decompression with intravenous dexamethasone 8 mg	2 (9)
Nasogastric decompression with fleet enema delivered via placement of a urinary catheter into the stoma	1 (5)

In the operative group of patients, four patients had a hybrid approach including laparoscopy and parastomal (circumferential stomal incision) approach. Three patients had laparotomy for repair and the same number of patients had a combined midline laparotomy and parastomal approach. One patient underwent a parastomal approach only (Table 5).

**TABLE 5:** Description of Operative techniques.

Variables	N (%)	Details
Laparoscopic only	2 (9)	Laparoscopic division of adhesions and parastomal hernia repair with Symbotex mesh to close defect (n = 1), Laparoscopic division of adhesions and parastomal hernia repair with Parietene mesh fashioned around colostomy and Symbotex mesh secured to anterior abdominal wall (n = 1)
Hybrid approach (Laparoscopic and Parastomal approach)	4 (18)	Laparoscopic adhesiolysis and reduction of parastomal hernia with open parastomal approach to resect hernia sac and placement of Symbotex mesh, followed by laparoscopic Sugabaker repair (n = 2), laparoscopic division of adhesions, open mobilisation of parastomal hernia, SMART procedure with prolene mesh and symbotex mesh for laparoscopic closure of hernia defect (n = 1), laparoscopic assisted adhesiolysis and reduction of hernia with parastomal approach for refashioning of stoma (n = 1)
Midline approach only	3 (14)	Laparotomy open adhesiolysis, reduction of parastomal hernia and closure of defect using biological mesh (n = 1), Laparotomy, transection of ileostomy for reduction of hernia and excision of sac, adhesiolysis, closure of defect with Permacol mesh and formation of new ileostomy (n = 1), Laparotomy open adhesiolysis, transverse colectomy, reversal of Hartmanns procedure, biomesh to transversalis fascia plane and repair of ventral hernias, intraoperative botox injection (n = 1)
Midline + Parastomal (circumferential stomal incision) approach	3 (14)	Laparotomy open adhesiolysis with parastomal incision to moblise stoma, closure of stoma, reduction of hernia, small bowel resection and anastomosis, closure of hernia defect and resiting of stoma (n = 1), Laparotomy open adhesiolysis with open parastomal incision to reduce hernia and primary closure of defect (n = 1), Laparotomy with extensive open adhesiolysis, open parastomal hernia incision for refashioning and resiting of stoma, biological mesh for closure of abdominal wall (n = 1)
Parastomal approach (circumferential stomal incision) only	1 (5)	Stoma mobilized from parastomal hernia, resection of ileostomy and anastomosis (reversal) and primary closure of defect (n = 1)
Resection of small bowel	4 (18)	
Resection of conduit	4 (18)	Resection colostomy conduit (n = 4)
Relocation of stoma	4 (18)	
Use of mesh	9 (41)	
Position of mesh		
Stoma site	4 (18)	
Abdominal wall	3 (14)	
Both	2 (9)	
Type of mesh used		
Bio-absorbable mesh	5 (23)	
Synthetic mesh	4 (18)	
Reversal of stoma	2 (9)	
Component separation	1 (5)	Tranversus abdominal release (n = 1)
Botox injection	2 (9)	Botox 300 units intramuscularly given intraoperatively (n = 1), Botox 300 units intramuscularly given postoperatively (n = 1)

Four patients required small bowel resection and the same number of patients underwent resection of stoma. Four patients had their stoma re-sited. Mesh was used in nine cases; Of which, bio-absorbable mesh was used in six patients and three of patients had synthetic mesh placed.

Reversal of stoma was performed in two patients and one required component separation during the index operation. Two patients received 300 units of botulinum injections intramuscularly either intraoperatively or postoperatively.

Median LOS was 7 days (3–17). Out of the nine non-operative patients, seven have undergone an elective operation after the admission. One patient received botulinum injection prior to the elective repair [11]. The remaining two patients did not undergo an elective repair and are lost to follow-up. There was one death in the operative group due to sepsis from multi-organ failure.

In terms of complications, two patients with Claven-Dindo grade II required decompression for ileus and small bowel obstruction respectively. One patient required operative management for small bowel obstruction post-operatively and two patients required intensive care admission involving organ support for sepsis due to small bowel anastomosis leak and peristomal sepsis respectively. Five patients have PSH recurrence (Table 6).

**TABLE 6:** Outcomes of management.

Variables	N (%)	Further details
Recurrence	5 (23)	
Elective operation for non-operative patients	7 (32)	
Botox pre-op	1 (5)	
Morbidity (Claven dindo):		
2	2 (9)	Ileus needing decompression (n = 1), small bowel obstruction needing decompression (n = 1)
3b	1 (5)	Small bowel obstruction needing operative management (n = 1)
4	2 (9)	Small bowel anastomosis leak requiring ICU admission (n = 1), peristomal sepsis requiring ICU admission (n = 1)
5	1 (5)	Death

^a^ median, (IQR, 25th percentile-75th percentile).

## Discussion

Parastomal hernia is a very common complication following the formation of ostomy. Its incidence varies with the duration of follow-up, and has been shown to increase with time [1]. The definition of parastomal hernia is problematic; if radiological criteria are used then the rate is very high and if clinical criteria are used, the incidence lower [12]. Most PSHs detected on imaging remain asymptomatic. However significant number of patients eventually require a repair in an elective setting [13]. The data in this study reflects the acute presentation of parastomal hernia.

There is a paucity of literature on the management of emergency presentation of PSH. The number of emergency operations performed for PSH, in this study, is in keeping with the majority of the literature as observed in studies from Sweden [14] with 22 emergency cases reported in a 10-year study, Spain [15] 24 cases in 10-year study and 7 cases in 5-year study from the United Kingdom [16]. A Danish study incorporating figures from their national data registry reported 169 emergency PSH repairs [17]. A recent US study based on Medicare data reports on 6658 emergency PSH repairs in older patients aged 65 and above from 2007 to 2015 [7]. Some of these studies lack more elaborate details on the description of management and outcomes of emergency PSH.

Our study has also demonstrated that the emergency presentation of PSHs is relatively uncommon over a decade in an Australian tertiary referral centre. More than half of the patients underwent surgical intervention during the index admission. Non-operative patients were managed with being nil by mouth and nasogastric tube decompression. Of the nine patients managed non-operatively, seven went on to have elective repair. Indeed 90% of this Australian cohort had surgical repair. Interestingly, a multicentre retrospective Dutch study on the non-operative management of PSH suggested that non-operative management of PSH could be appropriate in the elective setting [18]. Based on this study, patients who were managed non-operatively should have an elective repair in a semi-urgent timeframe to avoid the co-morbidity and mortality associated with emergency PSH repair.

The presence of a midline incisional hernia in patients with PSH is not uncommon and it adds significant complexity to the approach. It is surprising that a midline hernia was only present in one of our parastomal hernia emergency presentations. In our series, various surgical approaches were used, with a hybrid of laparoscopic and parastomal approach being the most common, followed by a midline approach or combined midline/parastomal approach. A parastomal approach was favoured in 16 cases in a study in Spain [15] while there were 3,433 patients in the large American data set [7], compared to only one patient in our study. In terms of concomitant midline hernia repair, the Spanish study had eight patients, while five patients needed simultaneous incisional hernia repair. A small number of 212 cases in the American study were managed with minimally invasive techniques [7]. With the right skill set, our data suggests a laparoscopic or hybrid/laparoscopic can technique be used in the emergency setting, with potentially less pain and a quicker recovery. This is also supported with data from a nationwide Danish study that showed an increase in emergency laparoscopic repairs at 72% and a steady reduction in open repairs [17].

In addition to the various PSH repair approaches, stoma relocation is an alternative option. Four patients received a relocation of stoma in this study *versus* 12 patients in a similar study [15]. Rubin et al. suggest that stoma relocation is better than fascial repair [3], however more recent studies have reported high recurrence rates of up to 76% at the new site [13]. Baxter and colleagues have shown in their study that stoma relocation is associated with higher odds of rehospitalisation, reoperation and mortality [7]. However, the risk of reoperation was significantly lower at 5-year follow-up outcome for stoma reversal. When deciding for stoma relocation, it should be kept in mind that the new trephine may need to be created at a larger size to accommodate the potentially oedematous conduit which then predisposes the patient to have higher recurrence rates at the new site. The old PSH site still needs repair as well.

Reversal of stoma in the emergency setting has not been commonly reported in current literature but this study had the same number of patients who received a reversal of stoma as Verdaguer (2020) [15]. Baxter et al. reported 24% of emergency PSH repairs had ostomy reversal [7]. Unfortunately, a reversible stoma is usually not available. For ileal urinary conduits, ostomy relocation is usually not be possible due to limitations of the retroperitoneal attachment of the uretero-ileal anastomosis. In cases of temporary diverting loop stomas that present with incarcerated PSH, reversing the stoma should be considered unless clinically contraindicated. For an end colostomy, from Hartmann’s procedure, reversal in the emergency setting has balance risk of the overall patient’s comorbidities, haemodynamic status, and a prolonged operation.

Four patients had mesh used in the repair of PSH with the majority having bio-absorbable mesh placed. In a similar study in Spain, 12 patients were reported to have mesh repair [15] (Table 7). Baxter and colleagues reported only 16% of emergency PSH repairs had mesh used. The low rate of mesh use in the emergency and contaminated settings is understandable. It is interesting that in cases with mesh use, for PSH repair, the risk of complication was lower (OR0.84, 95% CI 0.72–0.98) and risk of reoperations (HR 0.74, 95% CI 0.58–0.94) lower than without mesh. Newer evidence from complex abdominal wall reconstructions involving contaminated/dirty wounds and/or intestinal resections demonstrated the safety of bio-absorbable mesh [19]. This could be translated into PSH repairs.

**TABLE 7:** Comparisons with other studies on management of Emergency parastomal hernias.

	Ramli (2024)	Verdaguer (2020) [10]	Odensten (2020) [9]	Reali (2022) [12]	Baxter (2024) [5]
Study period and location	10 years, Australia	10 years, Spain	10 years, Sweden	5 years, United Kingdom	8 years, USA
Number of patients with acute parastomal hernia	22	24	22	7	6658
Percentage of Emergency operative management	59%	59%	31%	11%	100%(operative by definition; this based on item numbers)
Rate of laparoscopic approach	9%	50%	Not specified for emergency cases	0%	3% (including robotic)
Mesh repair	41%	50%	Not specified for emergency cases	Not reported	16%
Synthetic Mesh used	18%	50%	Not reported	Not reported	Not reported
Length of Stay	6 days (IQR (3.0–17.0)	Not reported	Not reported	Not specified for emergency cases	Not reported
Complication rate	23%	92%	18%	Not specified for emergency cases	62%
Recurrence rate	23%	42%	41%	18%	Not reported

It has also been recommended that the use of synthetic mesh in emergency cases, without gross enteric spillage, is associated with a significantly lower risk of recurrence, regardless of hernia size [20]. In addition, the use of dissolvable synthetic mesh has been reported as a feasible option even in contaminated complex abdominal wall hernias with post-operative infection rates of 9% [21].

It is known that emergency PSH repairs have a high rate of complication compared to elective surgery [2]. Our single mortality (5%) compares favourably to the literature 8%–25% in the emergency repair of PSH [2, 6]. Baxter et al. reports a mortality rate of 13% within the first 30-day post-operation in patients >65 years above undergoing emergency PSH repair. The 5-year mortality rate rose to 64%. The rate of complication in our study with 6 patients (23%) seems reasonable in comparison with a study reporting a higher rate of 92% [15]. Baxter et al. reported the 30-day complication rate of 62% and this persists over a 5-year period with a complication rate of 68% reported [7]. In terms of recurrence, our study reports 5 patients (23%) with recurrence of PSH with other studies ranging from 18%–42% (Table 7).

Our study has limitations. The retrospective nature of the study is likely to underestimate complications, both intraoperatively and post discharge. The number of cases is comparatively small but represents the real-world experience of a high-volume acute tertiary hospital emergency PSH over a period of 10 years in Australia. The lack of formal evidence and guidelines, in the literature, reflect the different approaches undertaken for emergency PSH management. The management of this group of patients requires expertise in both colorectal and abdominal wall reconstructive surgery. This complex problem requires further, prospective study.

## Conclusion

The majority of acute PSH presentations require operative intervention, be that emergency or elective surgery. There are considerable recurrence rates. The approach to PSH repair in the acute setting needs to be individualised as various techniques are applicable; the use of mesh should be considered. Further studies are required to assist with the development of guidelines on this important topic.

## Data Availability

The raw data supporting the conclusions of this article will be made available by the authors, without undue reservation.
